# Genome editing toward biofortified soybean with minimal trade-off between low phytic acid and yield

**DOI:** 10.1007/s42994-024-00158-4

**Published:** 2024-05-23

**Authors:** Wenxin Lin, Mengyan Bai, Chunyan Peng, Huaqin Kuang, Fanjiang Kong, Yuefeng Guan

**Affiliations:** 1 Sanya Institute of China Agricultural University, Sanya, 572000 China; 2 College of Agronomy, China Agricultural University, Beijing, 100193 China; 3 Guangdong Provincial Key Laboratory of Plant Adaptation and Molecular Design, Innovative Center of Molecular Genetics and Evolution, School of Life Sciences, Guangzhou University, Guangzhou, 510006 China; 4 College of Life Sciences, Fujian Agriculture and Forestry University, Fuzhou, 350002 China

**Keywords:** Genome editing, Phytic acid, Soybean, Agronomic traits, CRISPR/Cas9

## Abstract

Phytic acid (PA) in grain seeds reduces the bioavailability of nutrient elements in monogastric animals, and an important objective for crop seed biofortification is to decrease the seed PA content. Here, we employed CRISPR/Cas9 to generate a PA mutant population targeting PA biosynthesis and transport genes, including two *multi-drug-resistant protein 5* (*MRP5*) and three *inositol pentose-phosphate kinases* (*IPK1*). We characterized a variety of lines containing mutations on multiple *IPK* and *MRP5* genes. The seed PA was more significantly decreased in higher-order mutant lines with multiplex mutations. However, such mutants also exhibited poor agronomic performance. In the population, we identified  two lines carrying single mutations in *ipk1b* and *ipk1c*, respectively. These mutants exhibited moderately reduced PA content, and regular agronomic performance compared to the wild type. Our study indicates that moderately decreasing PA by targeting single *GmIPK1* genes, rather than multiplex mutagenesis toward ultra-low PA, is an optimal strategy for low-PA soybean with a minimal trade-off in yield performance.

**Supplementary Information:**

The online version contains supplementary material available at 10.1007/s42994-024-00158-4.

Dear Editor,

Soybean (*Glycine max*) is recognized as a major source of plant-based protein for human diets and feedstocks (Montgomery 2003). Soybean contains phytic acid (PA), also referred to as D-myo-inositol-1,2,3,4,5,6-hexakisphosphate, or InsP6 (Gillman et al. 2009), which is the main form of stored inositol and phosphate. In soybean seeds, PA can count for 4–5% of the seed dry weight, and 50% of the seed total phosphorus content (Oatway et al. 2001). Having six phosphate groups, PA belongs to a class of metal-chelating compounds that may bind to iron, zinc, calcium, magnesium, and potassium, among other metal cations (Raboy 2009). During seed germination, the PA complex releases metal cations and provides energy to support seedling growth. However, PA is considered a non-negligible anti-nutritional factor. PA lowers the absorption and utilization of minerals (e.g., iron, zinc, calcium) and negatively affects human health for people subsisting on grain-based diets, mainly in developing countries (Zhou and Erdman 1995). In addition, livestock after feeding may excrete significant PA salts into nature, causing environmental issues of phosphorus pollution (Sharpley et al. 1994). Therefore, an important objective for crop seed biofortification is to decrease the seed PA contents. It was estimated that low-PA soybean potentially provides 5-billion-dollar worth value, as a result of increased P availability and biofortification (Raboy 2020).

Low-phytic acid (*lpa*) soybean varieties with decreased PA content were generated in natural mutation, mutagenesis breeding, and hybridization (i.e., CX1834 (Oltmans et al. 2004) and *Gm-lpa*-TW-1 (Yuan et al. 2007)). The major genes responsible for the *lpa* germplasm include myo-inositol phosphate synthase (MIPS) (Yuan et al. 2017), the inositol pentose-phosphate kinase gene (*GmIPK1a*) in PA biosynthesis (Yuan et al. 2012), and the vital multi-drug-resistant protein 5 gene (*GmMRP5a*) involved in PA transport (Gillman et al. 2009). Mutants in *GmMIPS1* exhibited low PA yet tend to exhibit low yield performance, whereas *gmipk1a* and *mrp5a* mutants were shown to less affect agronomic traits (Song et al. 2022). Although the *lpa* varieties can provide benefits to the nutritional quality and to the environment, it remains challenging to develop high-yielding low-PA varieties due to a lack of germplasms. Moreover, the trade-off between low PA and yield traits in soybean remains elusive.

In this study, our objective was to generate a varieties of new *lpa* mutants by multiplex mutagenesis using CRISPR/Cas9, and characterize biofortified soybean with a minimal trade-off between low-phytic acid and yield. We first characterized the *GmIPK1* and *GmMRP5* family genes, and selected seed-specifical expressed gene members for genome editing. We identified three *GmIPK1* homologs expressed predominantly in soybean seeds (https://phytozome-next.jgi.doe.gov/), namely *GmIPK1a* (*Glyma.14G072200*), *GmIPK1b* (*Glyma.04G030000*), and *GmIPK1c* (*Glyma.06G030100*). Two seed-enriched *GmMRP5* genes, *GmMRP5a* (*Glyma.03G167800*) and *GmMPR5b* (*Glyma.19G169000*) were also identified. We designed two constructs using the pGES401 vector, namely KO-PA-V1 targeting the IPK1 genes and the KO-PA-V2 targeting the MRP5 genes (Fig. 1A). To test the potential of multiplex mutants in lowering PA content, we transformed KO-PA-V1 and KO-PA-V2 by employing a pooled transformation method (Li et al. 2019). Among 35 T0 transgenic lines, we identified 8 plants with only KO-PA-V1, 17 plants with only KO-PA-V2, and 10 plants with both constructs. Among these lines, we characterized various mutant types containing mutations on *GmIPK1* genes, *GmMRP5* genes, or multiplex mutants on multiple *GmIPK1* and *GmMRP5* genes. In the T2 progeny, we obtained and investigated several homologous mutants with various frameshift mutations on target genes (Fig. S1), including the single mutants *gmipk1b*^*194D*^ (designated *lpa*-1), *gmipk1c*^*1I*^ (*lpa-*2), and *gmmrp5b*^*4D*^ (*lpa-*3); double mutant *gmmrp5a*^*1I*^*/gmmrp5b*^*4D*^ (*lpa*-4); triple mutant *gmipk1a*^*1I*^*/gmmrp5a*^*703D*^*/gmmrp5b*^*4D*^ (*lpa*-5); and quadruple mutants *gmipk1b*^*1I*^*/gmipk1c*^*1I*^*/gmmrp5a*^*703D*^*/gmmrp5b*^*2D*^ (*lpa*-6) and *gmipk1a*^*1I*^*/gmipk1b*^*1I*^*/gmmrp5a*^*1I*^*/gmmrp5b*^*4D*^ (*lpa*-7) (Fig. 1B). We further analyzed the predicted off-target effects in the T2 generation among the multiple mutant lines, and no off-target mutations were detected (Fig. S2).

**Fig. 1:**
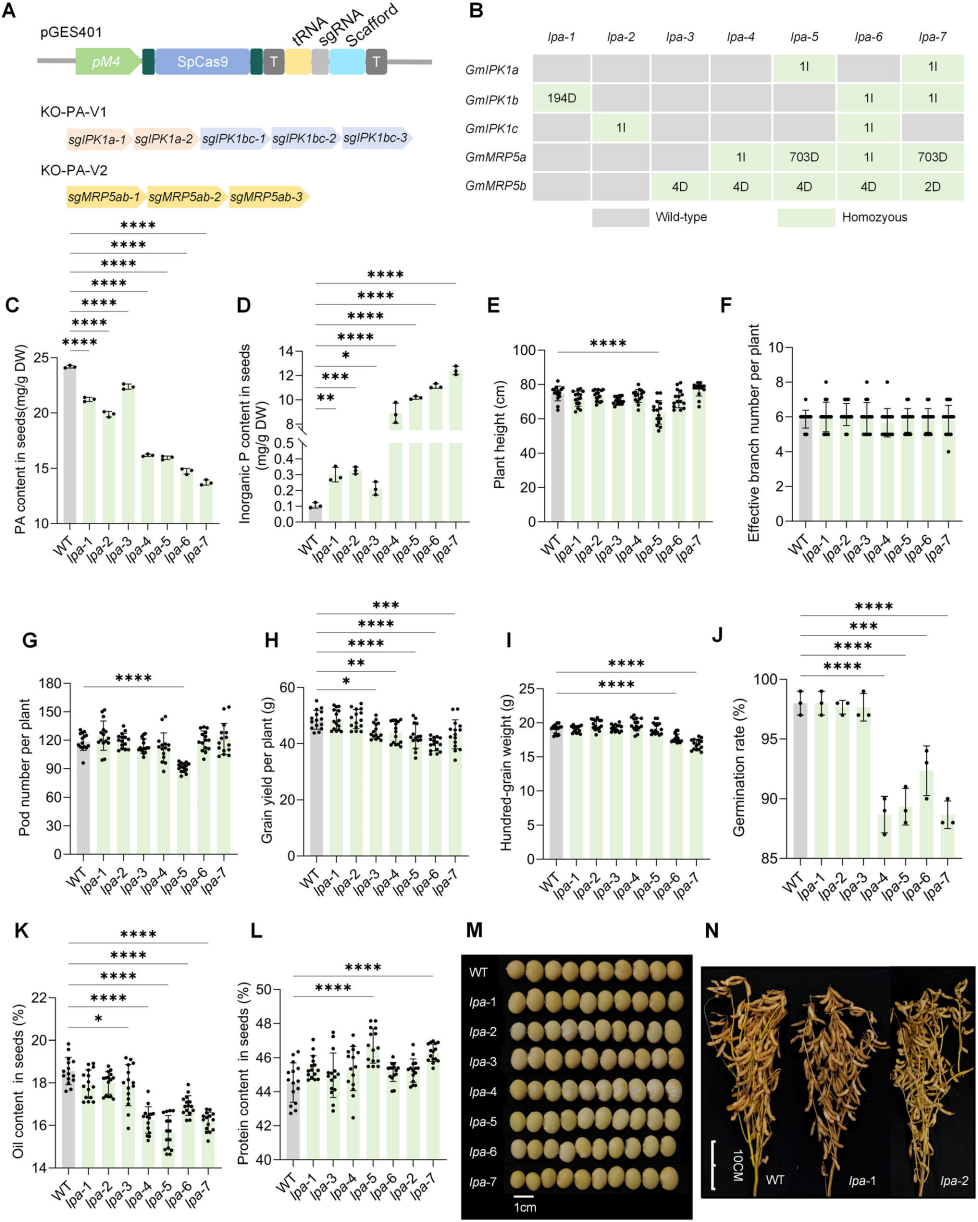
Generation and characterization of the PA mutant population in HC6. **A** Architecture of the pGES401 vector, and the sgRNA cassettes constructed on the genome editing plasmid, KO-PA-V1 and KO-PA-V2, respectively. **B** Genotype of the homozygous lines obtained in the T2 generation. **C** Determination of the PA content among the mutant lines,* n* = 3. **D** Determination of free phosphorus content in a variety PA mutant seeds, *n* = 3. **E-I** A comparative analysis of agronomic performance between PA mutants and the wild type HC6, including plant height (**E**), effective branch number per plant (**F**), pod numbers (**G**), grain yield per plant (**H**), and weight per hundred kernels (**I**). Significant differences were denoted by asterisks (*P*-values were determined by the two-way ANOVA test: *P < 0.05, **P < 0.01, ***P < 0.001, ****P < 0.0001), *n* = 15. **J** The germination rate of mature seeds exhibiting various mutations was compared to the baseline germination rate observed in the wild type, *n* = 3. **K**,**L** Determination of oil (**K**) and protein (**L**) content. **M **Seed size and shape of the PA mutant population lines and WT, *n* = 10. **N **Plant morphology of *lpa-1*, *lpa-2* and WT at harvest

We next determined the PA content of these mutants; these results showed a decreasing trend of PA content in all the mutants compared to the wild-type seeds. Of these, the PA in *lpa-*1 declined by 12.3%, *lpa-*2 decreased by 17.8%, and *lpa-*3 decreased by 7.5%. The PA content in higher-order mutants carrying *gmmrp5a* and *gmmrp5b* mutants was more drastically reduced by 33.1% in *lpa*-4, 34.0% in *lpa-*5, 39.1% in *lpa-*6 and the greatest decrease, 43.3%, in *lpa-*7 (Fig. 1C). PA and free phosphorus contents were negatively correlated. We next measured the free phosphorus content in the mutants, and the trend was the reverse of the PA content, with  an increase to 0.30 mg/g in *lpa*-1, 0.33 mg/g in *lpa*-2, 0.21 mg/g in *lpa-*3, 8.90 mg/g in *lpa*-4, 10.17 mg/g in *lpa-*5, 11.13 mg/g in *lpa-*6 and 12.41 mg/g in *lpa-*7 (Fig. 1D). Therefore, single mutations in the *GmIPK1b* or *GmIPK1*c genes moderately decreased PA content, whereas higher-order mutations significantly reduced PA and released large amount of free phosphorus.

To investigate the agronomic performance of these mutants, we assessed the plant height (Fig. 1E), number of effective branches per plant (Fig. 1F), pod numbers per plant (Fig. 1G), harvested grain weight per plant (Fig. 1H), hundred-grain weight (Fig. 1I), and germination rates of the mutant seeds (Fig. 1J). The single plant yield was not significantly affected in *lpa*-1 and *lpa*-2 carrying mutations in *gmipk1b* and *gmipk1c*, whereas *lpa*-3, with a single mutation in *GmMRP5b,* showed a slight reduction in grain production per plant. Moreover, the multiplex mutant lines *lpa-*4, *lpa-*5, *lpa-*6, and *lpa-*7 exhibited a notable reduction. The yield of both *lpa-*4 and *lpa-*5 showed a decline of approx. 10%, and the yield was about 42–43 g per plant (Fig. 1H). Additionally, it was noted that the *lpa-*6 and *lpa-*7 seeds displayed a decrease in 100-grain weight of more than 7%, accompanied by a reduction in seed size (Fig. 1I and M). The quadruple mutant lines, *lpa*-6, had significantly lower grain yield per plant, with a loss of 18% (Fig. 1H). In addition, we performed a small scale field trail in Sanya Hainan during the 2023 winter and the agronomic traits presented a similar tendency (Fig. S3).

Next, we evaluated the standard germination of PA mutants. These results were in line with prior research, and the germination rate of most multiplex mutants was significantly reduced (Fig. 1J). In contrast, *lpa-*1 and *lpa-*2 exhibited normal agronomic traits and a germination rate (98%) equivalent to the wild-type (Fig. 1J). At the harvest period, we assessed seed quality and observed an overall trend of decreasing oil content (Fig. 1K) and  improving protein content (Fig. 1L) in most PA mutant seeds, except for *ipk1b* and *ipk1c*. Finally, overall plant appearance of the *lpa-1* and *lpa-2* lines was not significantly difference from that of the WT (Fig. 1N). Therefore, single mutants in *ipk1b* and *ipk1c* tend to exhibit moderately decreased PA content and minimal yield penalty, whereas multiplex mutations of *IPK1*/*MRP* genes more dramatically decreased PA yet significantly negatively impacted agronomic performance.

Here, we used a multiplex CRISPR/Cas9-based genome editing approach to *IPK1*/*MRP5* genes, resulting in a variety of PA mutants. Our findings showed that the low-PA trait is negatively related with yield traits, in a dosage-dependent manner. The potential impact of reduced PA levels on seed germination might be due to the degraded PA serving as an energy source. In addition, germplasms carrying mutations on *MRP5* genes tend to exhibit negative effects on yield. In comparison with the ultra-low PA and comprised growth of multiplex mutants, single mutants in *IPK1* genes tend to moderately decrease PA with less affect on yield performance. Thus, by means of gene editing, we could reduce PA in mutants, release more free phosphorus, and increase the high bioavailability of soybeans to allow breeding toward “SMART CROPs” (Wang et al. 2024). In the future, using precision editing tools, such as base editing, might more precisely tune PA content for biofortified soybean with improved agronomic value (Bai et al. 2024).

## Materials and methods

### Plant material and growth conditions

Huachun 6 (WT), a soybean cultivar in south China, was utilized for the transformation. The mutant plants were grown in a greenhouse with a relative humidity of between 60 and 80% and 14/10 h of day/night temperature control of 27/25 °C. In addition, the yield evaluation of the PA homozygous mutants was completed in a transgenic field in Qingdao and Sanya, respectively, during which the planting conditions of the mutant and wild type were ensured to be consistent.

### Acquisition of PA genes editing transformants

CRISPR/Cas9 mutation pools of phytic acid in HC6 were performed using the protocol reported previously (Bai et al. 2020), and the genome editing vectors that targeted the PA genes, KO-PA-V1 and KO-PA-V2 (Fig. 1A), were constructed by the Goldengate assembly method. Stable transformants were obtained from soybean cotyledons by the *Agrobacterium*-mediated mix, also as described previously (Bai et al. 2020).

### DNA extraction and sequencing

To confirm the genotype of PA mutants, DNA from a soybean leaf two weeks after it germinated was extracted using the CTAB method. Sanger sequencing and Hi-TOM sequencing (Liu et al. 2019) were then used to find an exact mutation.

### Determination of PA content in seeds

Determination of total phosphorus in mature mutant seeds of T2 generation using Grace PA/total phosphorus kit (Suzhou Grace Biotechnology Co., Ltd) according to the manufacturer’s protocol. And this kit is modified from the original determination method(Heubner and Stadler 1919; Marolt and Kolar 2021). Briefly, 5 g of each mutant seeds was ground into powder and 100 mg of them was taken and extracted by shaking with acidic extractant for 2 h at room temperature. And further, the ferric chloride-sulfosalicylic acid color developer was discolored by the chelating effect of phytic acid. The phytic acid content is directly proportional to the degree of discoloration, and according to the manufacturer's instructions, the supernatant was tested for the decrease in absorbance at 500 nm to determine the phytic acid content of the samples.

### Evaluation of inorganic phosphorus in seeds

PA was indirectly assessed using the inverse relationship between inorganic phosphorus and PA in mature soybean seeds. The method was modified from the previous report (Raboy et al. 2000). The whole procedure consisted of mixing 1 mL of 10% (w/v) perchloric acid (PCA) with 0.5 g of mutant seed powder, which was then homogenized and crushed. Next, the homogenate was then diluted tenfold with 5% (w/v) PCA and refrigerated for 30 min. 10, 000 g centrifugation at 4 °C for 10 min was performed and the supernatant was transferred to a new tube. 2 mL of working solution was mixed with 1 mL of sample supernatant and incubated at 40 °C for 20 min. The reaction solution was cooled on ice and the absorbance was measured at 820 nm and converted to seed free phosphorus using a standard curve.

### Evaluation of germination rate between PA mutants

Germination rate is an important indicator of seed growth and development potential. Here, we modified the method published by international seed testing association (ISTA) to determine the standard germination rate of PA mutants. We used simultaneously harvested seeds in this experiment. From each PA mutant, 600 seeds were chosen at random, split into three replicates on average, and put in a germination box that was 80% wet. The standard germination rate was evaluated after 5 days of germination in an incubator at 25 °C.

### Determination of protein and oil content in seeds

Here, 15 individual plants of each PA mutant were randomly selected from the above materials that have been assessed for agronomic traits, and 20 g seeds were weighed for each strain. With the use of a MATRIX-I Fourier transform near infrared reflectance spectrometer (FT-NIRS) (Bruker Optics, Bremen, Germany), the protein and oil content tendency among mutants were determined.

### Supplementary Information

Below is the link to the electronic supplementary material.

Supplementary file1 (DOCX 2276 KB)

## Data Availability

All data supporting the findings of this study are available within the paper and supplementary information.
