# Supplementary material for: Genome editing toward biofortified soybean with minimal trade-off between low phytic acid and yield
Source: aBIOTECH. 2024 May 23;5(2):196–201. doi: 10.1007/s42994-024-00158-4 (PMC11224060; doi:10.1007/s42994-024-00158-4)
Supplement: Supplementary file 1 — Supplementary file1 (DOCX 2276 KB) [file 42994_2024_158_MOESM1_ESM.docx]

**Genome Editing Toward Biofortified Soybean with Minimal Trade-off Between Low-Phytic Acid and Yield**

Wenxin **Lin^1,2^,** Mengyan **Bai^3^,** Chunyan **Peng^4^,** Huaqin **Kuang^3^,** Fanjiang **Kong^3^,** Yuefeng **Guan^3*^**

^1^ Sanya Institute of China Agricultural University, Sanya 572000, China

^2^ College of Agronomy, China Agricultural University, Beijing 100193, China

^3^ Guangdong Provincial Key Laboratory of Plant Adaptation and Molecular Design, Innovative Center of Molecular Genetics and Evolution, School of Life Sciences, Guangzhou University, Guangzhou 510006, China

^4^ College of Life Sciences, Fujian Agriculture and Forestry University, Fuzhou 350002, China

*** Correspondence:** Yuefeng Guan ([Guan@gzhu.edu.cn](mailto:Guan@gzhu.edu.cn))

**Supplementary Information**

**Figure of contents**

[Fig. S1. Prediction of protein mutation types the protein domain. 3](#_Toc162005378)

[Fig. S2. Analysis of off-target effects in T2 generation at each targeting sites. 4](#_Toc162005379)

[Fig. S3. Agronomic trait performance between different PA mutants and wild type in Hainan. 7](#_Toc162005380)

# Fig. S1. Prediction of protein mutation types the protein domain.


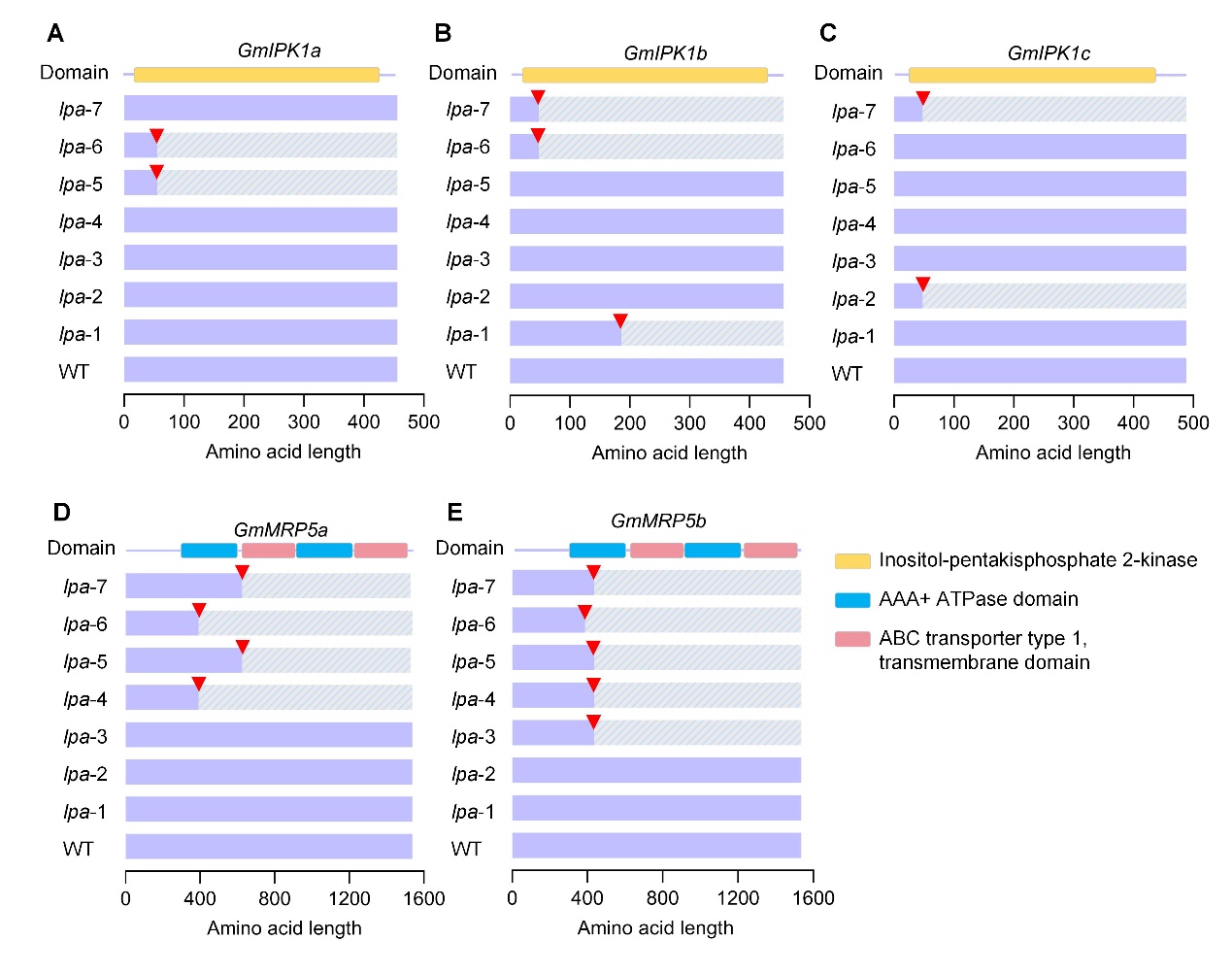


**Fig. S1** **Prediction of protein mutation types of different mutant lines and comparison with wild-type protein and the protein domain.**

The light purple area stands for the wild-type protein. The gray with blue shading sequences represent the frameshift mutation regions and the red triangle show the mutation sites of each protein. In addition, the light yellow areas stand for the Inositol-pentakisphosphate 2-kinase domain, the blue areas stand for the AAA+ ATPase domain and the pink areas stand for the transmembrane domain.

# Fig. S2. Analysis of off-target effects in T2 generation at each targeting sites.


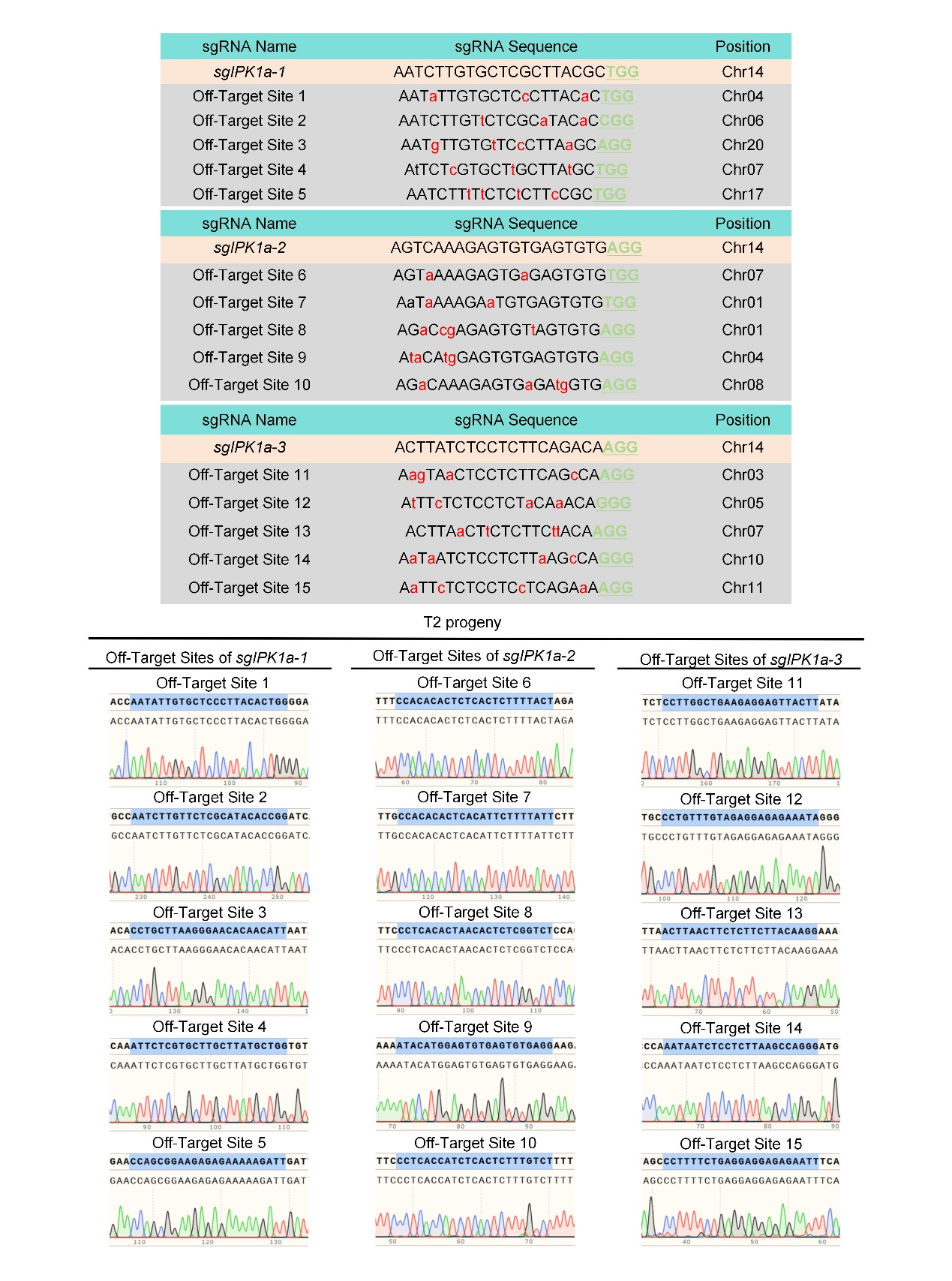


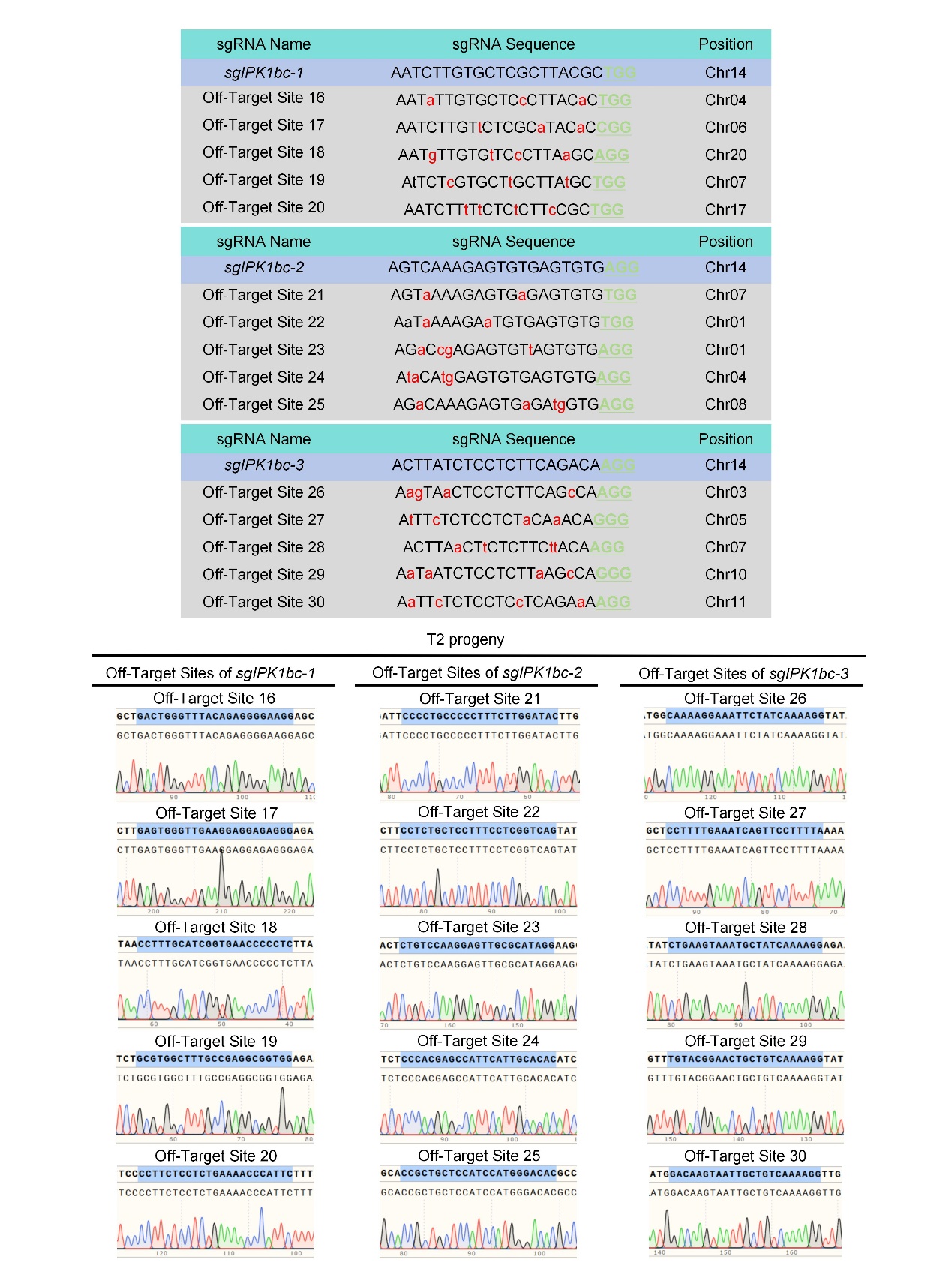


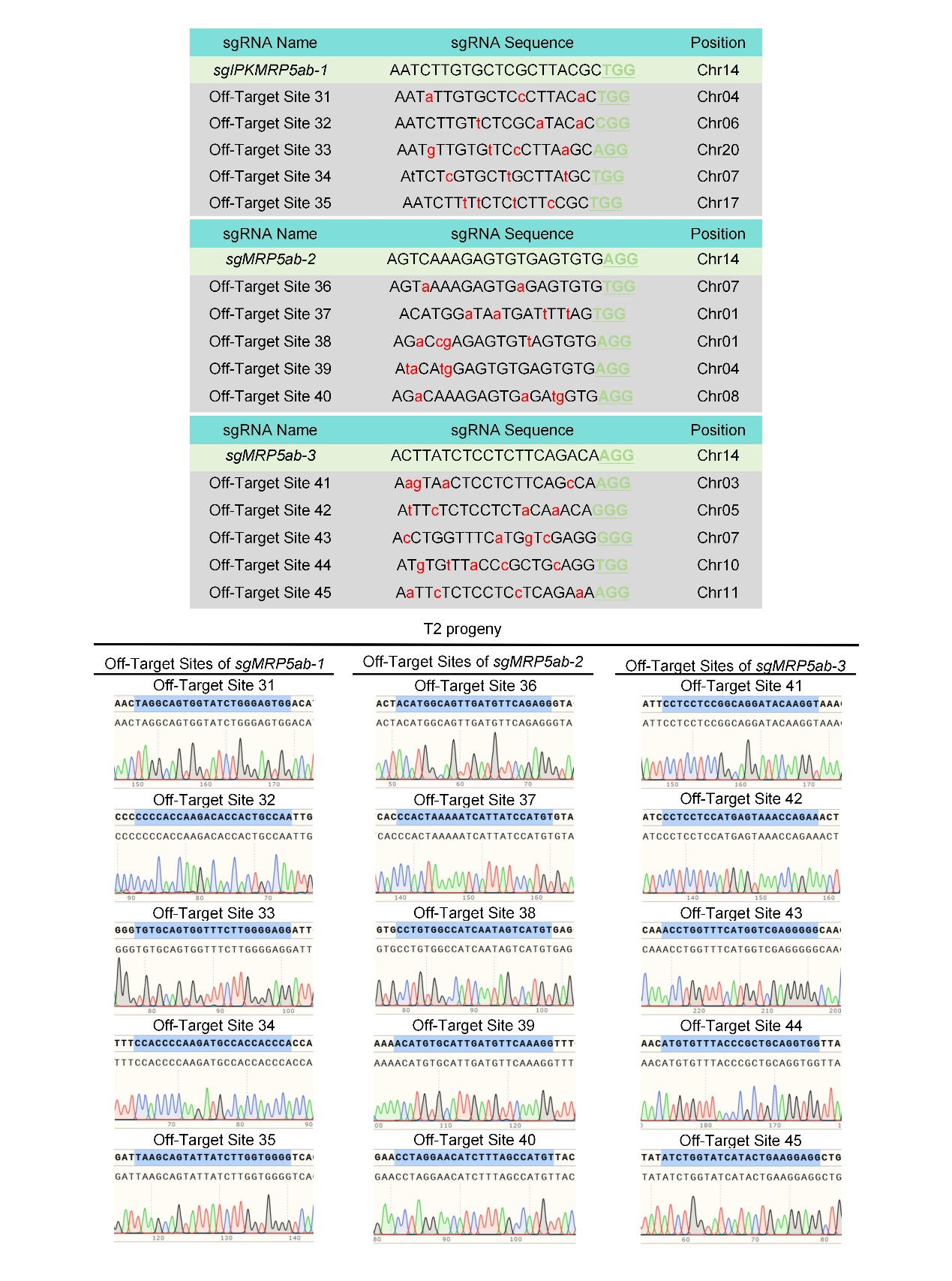


**Fig. S2 Analysis of off-target effects in T2 generation at each targeting sites**, including *sgIPK1a-1*, *sgIPK1a-2*, *sgIPK1a-3*, *sgIPK1bc-1*, *sgIPK1bc-2*, *sgIPK1bc-3*, *sgMRP5ab-1*, *sgMRP5ab-2* and *sgMRP5ab-3*, by Sanger sequencing respectively.

# Fig. S3. Agronomic trait performance between different PA mutants and wild type in Hainan.


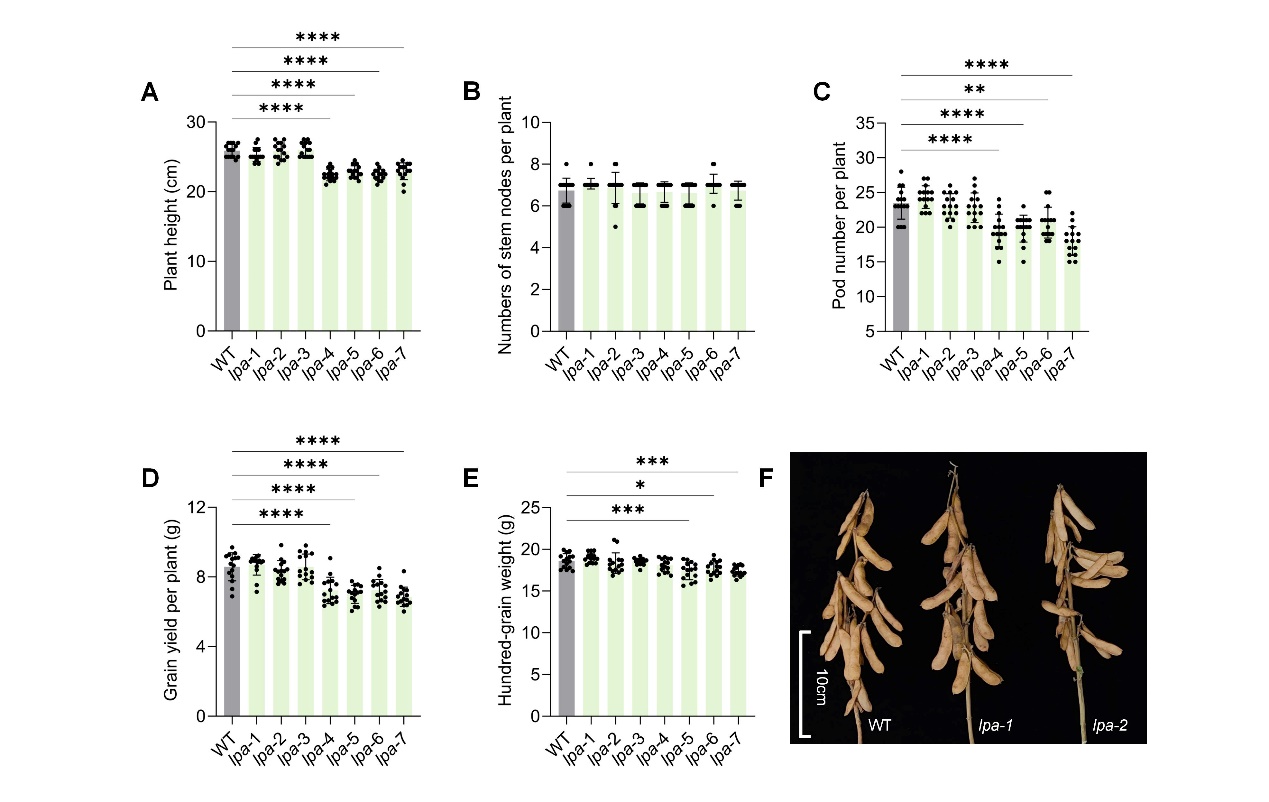


**Fig. S3 Agronomic trait performance between different PA mutants and wild type in Hainan,** including **A)** the plant height, **B)** the number of stem nodes per plant, **C)** pod numbers, **D)** grain yield per plant, and **E)** weight per hundred kernels. Significant differences were denoted by asterisks (*P*-values were determined by the two-way ANOVA test: **P*< 0.05, ***P* < 0.01, ****P*< 0.001, *****P*<0.0001), *n*=15. **M)** Plant morphology of *lpa-*1, *lpa*-2 and WT at harvest.
